# Cryo-EM Visualization of Lipid and Polymer-Stabilized Perfluorocarbon Gas Nanobubbles - A Step Towards Nanobubble Mediated Drug Delivery

**DOI:** 10.1038/s41598-017-13741-1

**Published:** 2017-10-18

**Authors:** Christopher Hernandez, Sahil Gulati, Gabriella Fioravanti, Phoebe L. Stewart, Agata A. Exner

**Affiliations:** 1 Department of Biomedical Engineering, Case Western Reserve University, Cleveland, Ohio, USA; 2 Department of Pharmacology, Case Western Reserve University, Cleveland, Ohio, USA; 3 Cleveland Center for Membrane and Structural Biology, Case Western Reserve University, Cleveland, Ohio, USA; 4 Department of Radiology, Case Western Reserve University, Cleveland, Ohio, USA

## Abstract

Gas microbubbles stabilized with lipids, surfactants, proteins and/or polymers are widely used clinically as ultrasound contrast agents. Because of their large 1–10 µm size, applications of microbubbles are confined to the blood vessels. Accordingly, there is much interest in generating nanoscale echogenic bubbles (nanobubbles), which can enable new uses of ultrasound contrast agents in molecular imaging and drug delivery, particularly for cancer applications. While the interactions of microbubbles with ultrasound have been widely investigated, little is known about the activity of nanobubbles under ultrasound exposure. In this work, we demonstrate that cryo-electron microscopy (cryo-EM) can be used to image nanoscale lipid and polymer-stabilized perfluorocarbon gas bubbles before and after their destruction with high intensity ultrasound. In addition, cryo-EM can be used to observe electron-beam induced dissipation of nanobubble encapsulated perfluorocarbon gas.

## Introduction

Shell-stabilized gas microbubbles (MBs) are frequently utilized as ultrasound contrast agents and vehicles for drug delivery. The latter application is especially attractive for cancer chemotherapy, as MBs can be externally triggered to release their cargo, enhancing drug distribution within the tumors and improving therapy outcomes^1–3^. MB oscillations and eventual dissipation or collapse in the ultrasound field, along with related bioeffects, have been the subject of extensive study^4–6^. Most routinely investigated MBs consist of a single layer of self-assembled phospholipids that are oriented through hydrophobic forces around a gas core (usually a perfluorocarbon). MB dissipation under ultrasound has been linked to two different mechanisms: acoustic dissolution and fragmentation into smaller bubbles^7,8^.

Because MBs have diameters on the order of 1–10 µm, their applications in cancer drug delivery are constrained to the vasculature. Accordingly, to broaden the scope of applications, there has been much recent interest in the creation of sub-micron bubble populations. These nanobubbles, which ideally would have diameters of 100–300 nm, have the potential to move beyond the vasculature into the tumor parenchyma. When placed in close proximity to tumor cells, nanobubbles could target specific biomarkers on the cell surface, be internalized into cells, and employ the same cavitation-based effects directly to the tumor cell membrane. These enhanced capabilities of nanobubbles could potentially lead to even greater therapeutic benefits.

Recent reports have demonstrated some of the benefits associated with nanobubbles in applications ranging from molecular imaging to therapeutic delivery^9–14^. However, due to the small size of nanobubbles, little is known about their morphology or response to ultrasound, as traditional techniques such as high-speed camera capture of bubble oscillations^15^ have an insufficient spatial resolution. Other techniques such as scanning electron microscopy (SEM)^16^ cannot directly visualize the fragile bubbles, and any acquired images would likely show ‘deflated’ bubbles that no longer contain the gas core^17^.

Cryo-electron microscopy (cryo-EM) is playing an increasingly important role in the visualization of lipid- and polymer-based nanoparticles^18^. Some recent examples include Doxil, a pegylated liposome that encapsulates doxorubicin^19^, liposomes with incorporated clusters of superparamagnetic iron oxide nanoparticles^20^, mixed polymer brush-grafted silica nanoparticles^21^, and virus-like particle−polymer conjugates^22^. Cryo-EM offers the advantage of being able to visualize a specimen in a near-native, albeit frozen, state. This feature has made cryo-EM a key validation tool for examining the structural integrity of nanoparticles.

In this study, we report the visualization of gas nanobubbles stabilized with lipid/polymer hybrid shells using cryo-EM. We investigated the effect of polymer crosslinking on nanobubble morphology and the response of nanobubbles to sonication and electron-beam driven dissipation. This study demonstrates that cryo-EM can be a powerful characterization tool for gas nanobubbles, which are too fragile for imaging by SEM or by negative-stain transmission electron microscopy (TEM). We anticipate that cryo-EM visualization will become a necessary tool for the structural validation of nanobubbles designed for future molecular imaging and drug delivery applications.

## Experimental

### Materials and methods

#### Lipids, polymers, and crosslinking agents

The lipids DPPC (1,2-dipalmitoyl-sn-glycero-3-phosphocholine), DPPA (1,2 dipalmitoyl-sn-glycero-3-phosphate), and DPPE (1,2-dipalmitoyl-sn-glycero-3-phosphoethanolamine) were obtained from Avanti Polar Lipids (Pelham, AL), and mPEG-DSPE (1,2-distearoyl-*sn*-glycero-3-phosphoethanolamine-N-[methoxy(polyethylene glycol)-2000] (ammonium salt)) was obtained from Laysan Lipids (Arab, AL). N, N-diethyl acrylamide (NNDEA), 2-Hydroxy-4′-(2-hydroxyethoxy)-2-methylpropiophenone (Irgacure 2959), and N, N-bis(acryoyl) cystamine (BAC) were purchased from Sigma Aldrich (Milwaukee, WI). Pluronic L10 was donated by BASF (Shreveport, LA).

#### Bubble formulation and nanobubble separation

Crosslinked nanobubbles (CL-NBs) were prepared as described by Perera *et al*.^23^. Briefly, nanobubbles were prepared by dissolving the lipids DPPC, DPPA, DPPE, mPEG-DSPE in chloroform in a 4:1:1:1 mass ratio. The solvent was then removed by evaporation and the lipids were hydrated in a solution containing 50 μL of glycerol and 1 mL of phosphate buffered saline (PBS) containing 0.06 wt.% Pluronic L10, and 0.5 wt.% Irgacure 2959 at 70 °C for 30 min. Following the hydration step, NNDEA and BAC were added and dissolved into the lipid solution and the vial was resealed before the air inside the vial was replaced with octafluoropropane (C_3_F_8_). Finally, the vial was agitated using a VialMix shaker (Bristol-Myers Squibb Medical Imaging, Inc., N. Billerica, MA) for 45 s, and irradiated at 254 nm using a UV lamp (Spectronics Co. Westbury, NY) for 30 min.

To formulate Pluronic (non-crosslinked) nanobubbles, the above mentioned lipid film was hydrated instead in a solution containing 50 μL of glycerol and 1 mL of PBS containing 0.06 wt. % Pluronic L10 at 70 °C for 30 min. Once hydrated, the air inside the vial was replaced with octafluoropropane (C_3_F_8_) and agitated using a VialMix for 45 s.

Nanobubbles were separated from microbubbles based on their buoyancy by centrifugation. The terminal velocity (ν_∞_) of a bubble in a liquid medium can be estimated by applying the force balance between the bubble buoyancy force and the Stokes drag on a particle^24^.

1 $${\upsilon }_{\infty }=\frac{1}{18}\frac{g{d}^{2}(\rho -{\rho }_{b})}{\mu }$$

where *d* is the bubble diameter, *g* is the gravitational acceleration, *μ* is the viscosity of the fluid medium (0.001 N∙s∙m^−2^), ρ and ρ_b_ are the densities of the fluid medium (1000 kg∙m^−3^) and bubble (8.17 kg∙m^−3^), respectively. According to this equation, a bubble larger than 0.7 µm should rise a distance of 0.5 cm or greater following centrifugation at 50∙*g* for five minutes. Care was taken to only collect samples below this distance.

#### Nanobubble characterization

Initial nanobubble size and concentration for each formulation was determined by using Nanosight nanoparticle tracking analysis (NTA) (Malvern Instruments, UK). Particle distributions before and after destruction with ultrasound were measured by Dynamic Light Scattering (DLS) using a Litesizer 500 (Anton Par, Austria). This DLS can measure particles between 0.3 nm and 10 μm and therefore should be capable of identifying the presence of structures from micelles to large lipid sheets. Samples for DLS and particle tracking were measured by diluting samples 1:1000 and 1:2000 with PBS at pH 7.4, respectively (n = 3). Detailed acoustic characterization of the nanobubbles and CL-NBs was previously reported^25^.

#### Ultrasound mediated nanobubble destruction

A 1.5 wt.% agarose phantom was prepared inside of a 6 well plate with a custom made rectangular insert. After gelling, the agarose phantom was removed from the six well plate, exposing the rectangular trough. The agarose phantom was fixed above the ultrasound transducer and the isolated nanobubbles were diluted by 1:100 in PBS and transferred to the trough. Bubble contrast was monitored using an AplioXG SSA-790A clinical ultrasound scanner (Toshiba Medical Imaging Systems, Otawara-Shi, Japan) equipped with a 12 MHz linear array transducer. System acquisition parameters were set to contrast harmonic imaging (CHI) with 12.0 MHz harmonic frequency, 0.1 mechanical index (MI), 65 dB dynamic range, and 70 dB gain. Bubbles were destroyed using the flash/replenish feature on the clinical ultrasound (10 flash cycles, 12 MHz harmonic frequency, 1.52 MI).

#### Cryo-EM imaging

Small aliquots (5 μL) of CL-NBs and non-crosslinked NBs at concentrations of 10^10^ particles/ml were applied to Quantifoil holy carbon EM grids (R2/2, 400 mesh; EMS) before and after ultrasound disruption. The sonicated samples were vitrified within 1 hour of sonication. The EM grids were glow-discharged for 30 seconds at 15 mA before sample application. Grids were then blotted and plunge-frozen into liquid ethane by using a manual plunger. Cryo-EM grids were imaged on a JEOL 2200FS transmission electron microscope (200 kV, FEG, in-column energy filter) equipped with a Tietz TVIPS 4k × 4k CMOS camera. Micrographs were collected with a defocus range of −4 to −4.5 µm and with a total electron dose of <60 e^−^/A^2^. Series acquisition showing electron beam damage to the nanobubbles involved a total electron dose of <900 e^−^/Å^2^ distributed over 45 frames. Individual frames were collected with 800 ms exposures resulting in a total acquisition time of over 40 s. Image analysis was performed with ImageJ software^26^.

#### Data availability

The datasets generated during and/or analyzed during the current study are available from the corresponding author on reasonable request.

## Results and Discussion

### Nanobubble production and characterization

**Figure 1:**
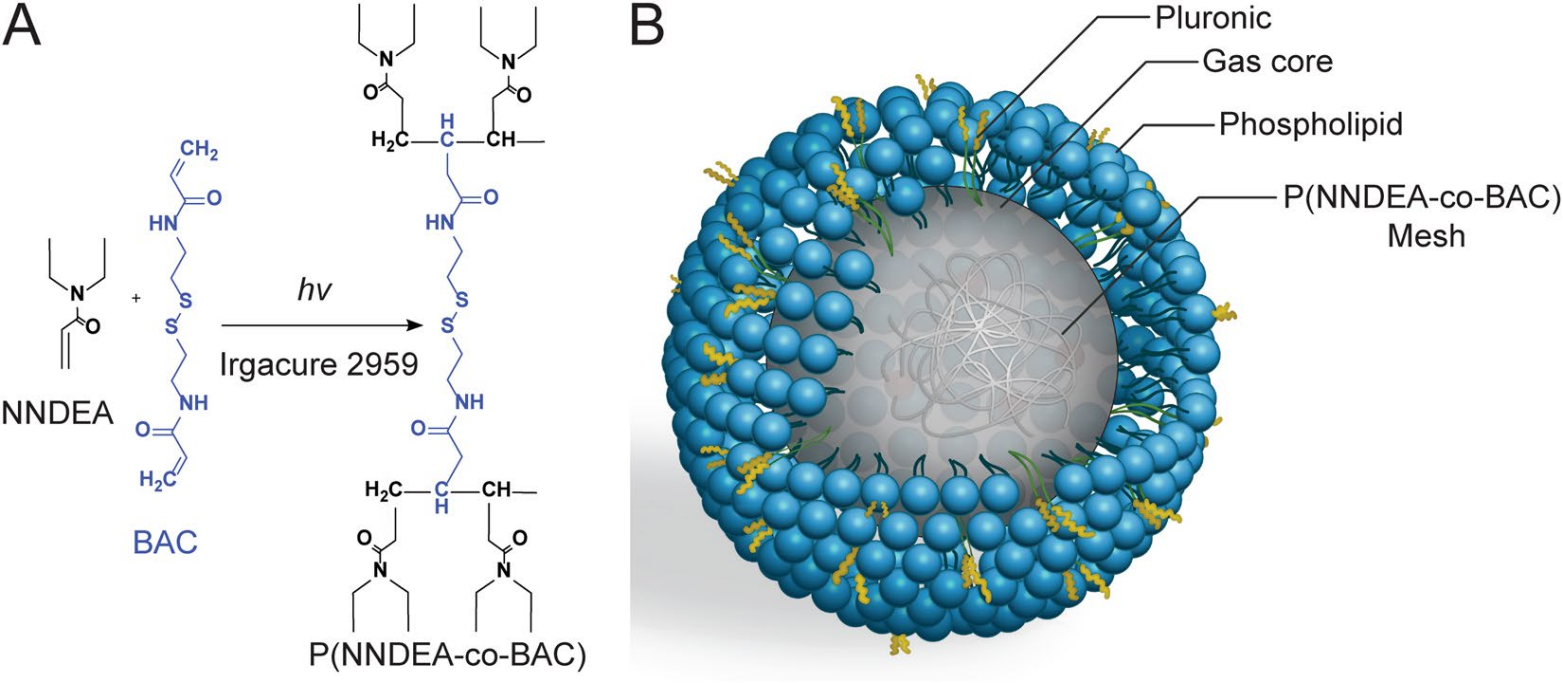
Production of crosslinked nanobubbles (CL-NBs). (**A**) UV irradiation is used to form a crosslinked network of the biodegradable polymer N,N-diethyl acrylamide (NNDEA) with cross-linker N,N-bis(acryoyl) cystamine (BAC). (**B**) Schematic diagram of a CL-NB. A P(NNDEA-co-BAC) crosslinked mesh stabilizes an outer pluronic/phospholipid layer enclosing a perfluorocarbon gas core. Illustration credit to Tiffany Yang.

Both non-crosslinked and crosslinked nanobubbles (CL-NBs) with perfluorocarbon gas cores were prepared for characterization. For CL-NBs, UV irradiation was used to form a crosslinked polymer mesh designed to stabilize the nanobubbles (Fig. 1). Nanoparticle tracking analysis of both types of nanoparticles revealed a more uniform size and a higher peak concentration for CL-NBs (2.5 × 10^11^ particles/ml) as compared to non-crosslinked nanobubbles (1.1 × 10^11^ particles/ml) (Fig. S1). The higher peak concentration for CL-NBs may indicate that the crosslinked polymer mesh enhances the stability of nanobubbles during centrifugation. Both formulations produced nanobubbles with mean diameters of ~140 nm and typical size ranges of 50–500 nm as determined by nanoparticle tracking.

**Figure 2:**
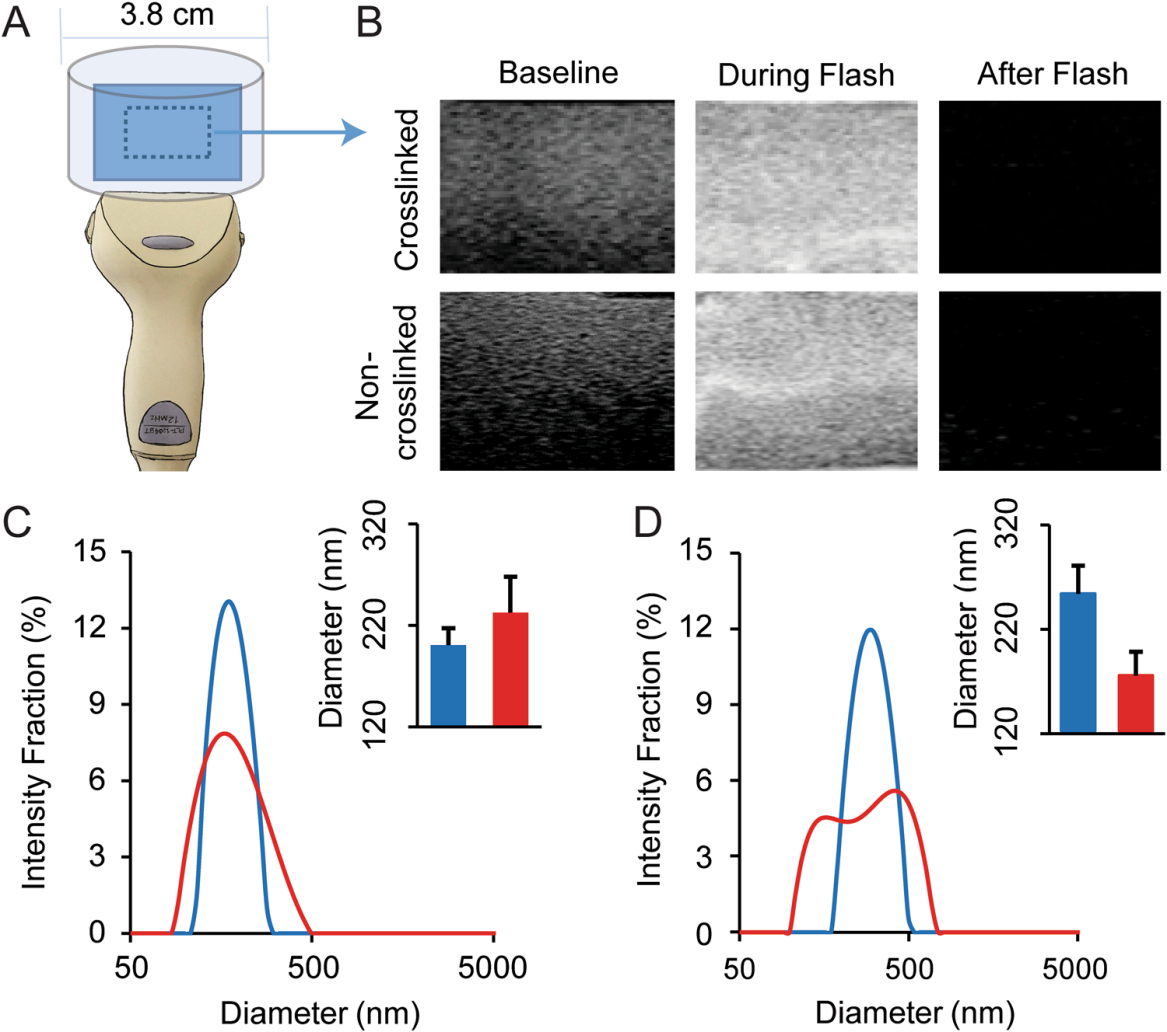
Ultrasound-driven nanobubble dissipation and size characterization. (**A**) Schematic of the ultrasound transducer (bottom) and agarose phantom/sample location (blue). (**B**) Ultrasound images of crosslinked and non-crosslinked NBs before, during and after a high-power ultrasound flash cycle. (**C**) Dynamic light scattering intensity-weighted size distributions of crosslinked NBs before (blue) and after (red) ultrasound destruction. (**D**) Dynamic light scattering intensity-weighted size distributions of non-crosslinked NBs before (blue) and after (red) ultrasound destruction. Insets in panels C and D show the mean intensity-weighted hydrodynamic diameters for each group.

To characterize the behavior of non-crosslinked and crosslinked nanobubbles during exposure to a high-power ultrasound, agarose phantoms with both types of nanobubble samples were created. A clinical ultrasound transducer was used to capture images before, during, and after the ultrasound flash cycle (Fig. 2A,B). Both types of nanobubble samples behaved similarly and a complete loss of ultrasound signal is evident for both samples following ultrasound application. Estimation of the size distribution of the nanobubbles before and after ultrasound destruction was obtained with dynamic light scattering (DLS). The DLS data show that the diameter of the CL-NBs remains similar before and after an ultrasound flash, with just a slight increase in the mean diameter from 200 +/−17 nm to 233 +/− 35 nm and a slightly broader size distribution (Fig. 2C). In contrast, the non-crosslinked NBs display a decrease in mean diameter from 296 +/− 12 nm to 252 +/− 51 nm after an ultrasound flash and their size distribution broadens more substantially.

### CryoEM visualization of nanobubbles

**Figure 3:**
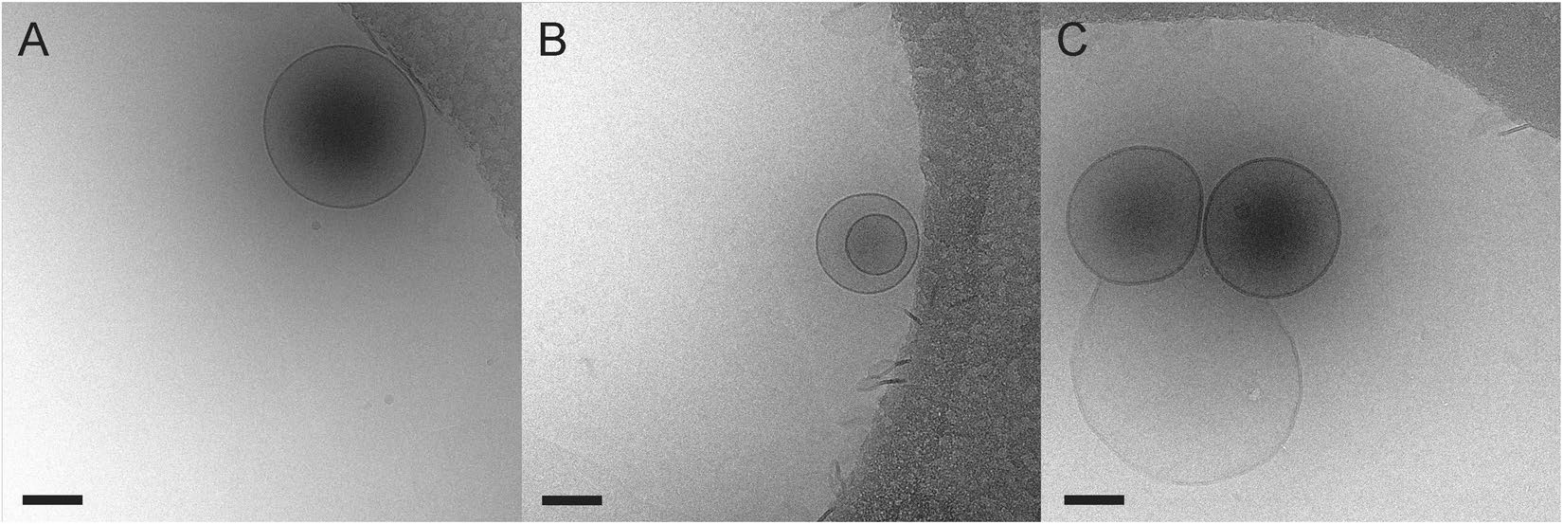
Cryo-EM images of CL-NBs. (**A**) A single CL-NB next to the carbon support layer of the EM grid (upper right-hand corner). (**B**) A larger nanobubble encapsulating a smaller nanobubble. (**C**) Two CL-NBs interacting with a larger malformed nanobubble (light center). Scale bars, 100 nm.

In order to visualize the nanobubbles, cryo-EM imaging techniques were employed. This approach involves flash freezing a concentrated sample on an EM grid in a cryogen. Then while keeping the sample grid at liquid nitrogen temperature, it is imaged with a low dose of electrons in a transmission electron microscope. Cryo-EM images were collected of crosslinked (Fig. 3) and non-crosslinked nanobubbles (Fig. S2). Both types of nanobubbles appear similar with an approximately spherical shape ranging in diameter from 100 to 500 nm, an outer monolayer, and an electron dense core. Many of the nanobubbles were observed next to the carbon support layer of the EM grid (Fig. 3A). This could be due to the presence of thicker vitreous ice around the edges of the holes. During the blotting step of cryo-EM grid preparation, the nanobubbles could have moved toward the edges of the hole in order to remain as hydrated as possible.

Since cryo-EM is a form of transmission electron microscopy, the acquired images are projection images and the observed density corresponds roughly to the mass in the particle. Typically, stains are not used in cryo-EM and image contrast is created by both amplitude contrast (arising from density variations between the particle and the surrounding frozen layer) and phase contrast (generated by collecting images slightly out of focus). We hypothesize that the dark cores observed in cryo-EM images of crosslinked and non-crosslinked nanobubbles are indicative of encapsulated octafluoropropane gas (molecular weight of 188 g/mol). We note that dark cores have recently been observed in cryo-EM images of a nanobubble formulation designed for enhanced macromolecular delivery to the retina^27^. A small percentage (<10%) of nanobubbles in our preparation are observed without the electron dense core and these are presumably empty or malformed nanobubbles (Fig. 3C).

**Figure 4:**
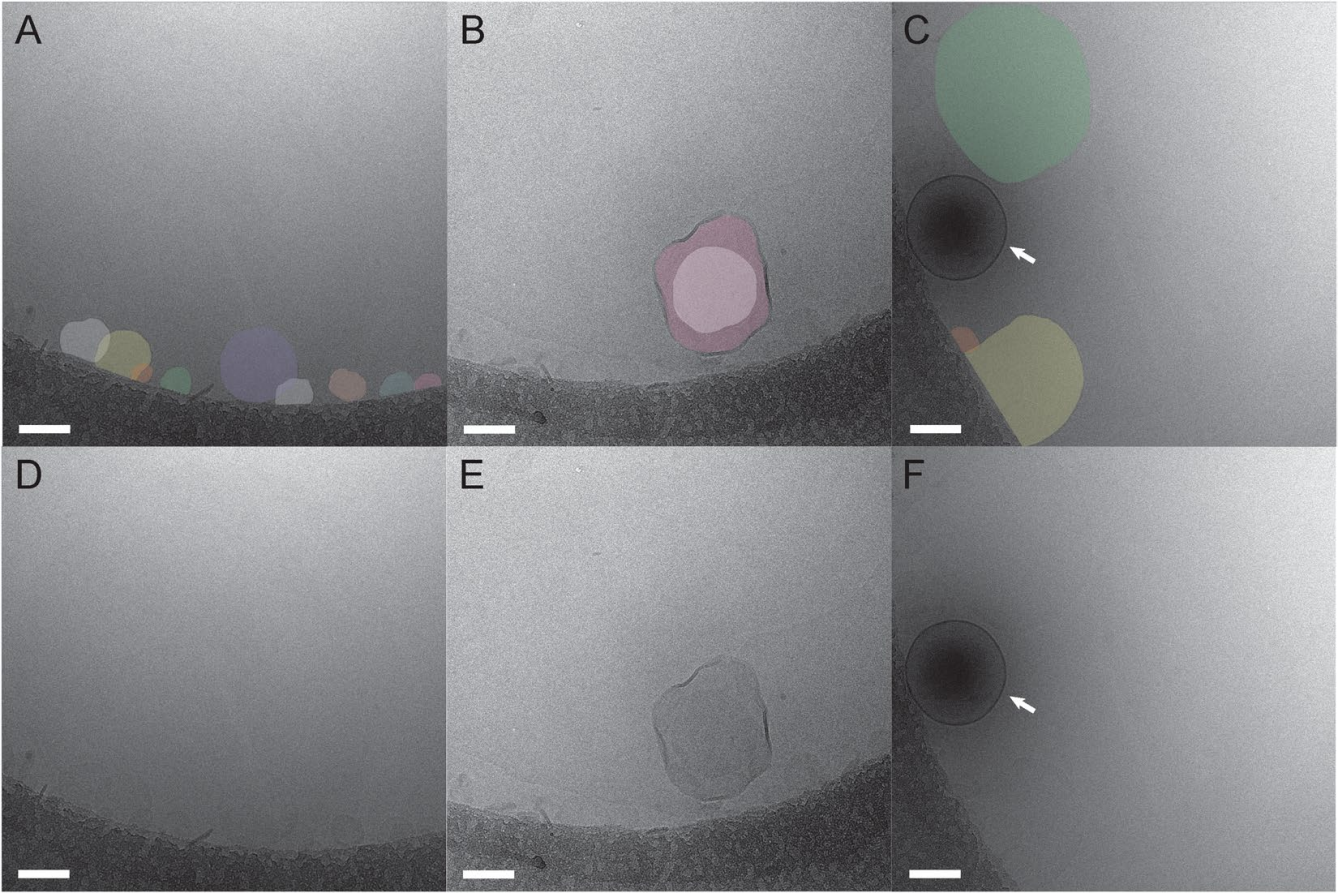
Cryo-EM images of sonicated CL-NBs. (**A–C**) Disrupted nanobubbles are shown colorized for better visualization. In panel A, multiple disrupted CL-NBs are observed along the carbon support layer of the EM grid. Occasionally, a multi-layered disrupted CL-NB is observed as in panel B. In panel C, one intact CL-NB is observed with a dense core (arrow). (**D–F**) Non-colorized versions of panels A-C. Scale bars, 100 nm.

If the dark cores do indeed represent electron density from the encapsulated gas, then exposure of the nanobubbles to an ultrasound flash should release the gas and the resulting disrupted nanobubbles should have lighter cores in cryo-EM images. To test this idea, we prepared cryo-EM grids of crosslinked and non-crosslinked nanobubbles after exposure to a high power ultrasound flash. As anticipated, cryo-EM images of ultrasound disrupted CL-NBs reveal that the vast majority of nanobubbles (>90%) have significantly less dense cores (Fig. 4). An occasional intact CL-NB with a dense core is observed (Fig. 4C) and these must have escaped or withstood the effects of the ultrasound. In fact, the image contrast of ultrasound disrupted CL-NBs is so low that the nanobubbles almost blend in with the surrounding frozen ice layer and colorization was used to aid visualization (Fig. 4A–C). The presence of some aggregates in cryo-EM images of sonicated CL-NBs (Fig. 4A) offers a possible explanation for the slight increase in mean particle diameter after sonication as measured with DLS (Fig. 2C). Comparing cryo-EM images of CL-NBs with and without prior exposure to ultrasound shows that the nanobubbles become slightly more irregular in shape and less spherical (Figs 3 and 4). Cryo-EM sample grids are flash frozen in a cryogen to produce vitreous, non-crystalline ice, and sample particles are normally well preserved during the vitrification process. Therefore, we conclude that a high power ultrasound flash partially disrupts the outer monolayer of CL-NBs allowing rapid release of the encapsulated gas.

**Figure 5:**
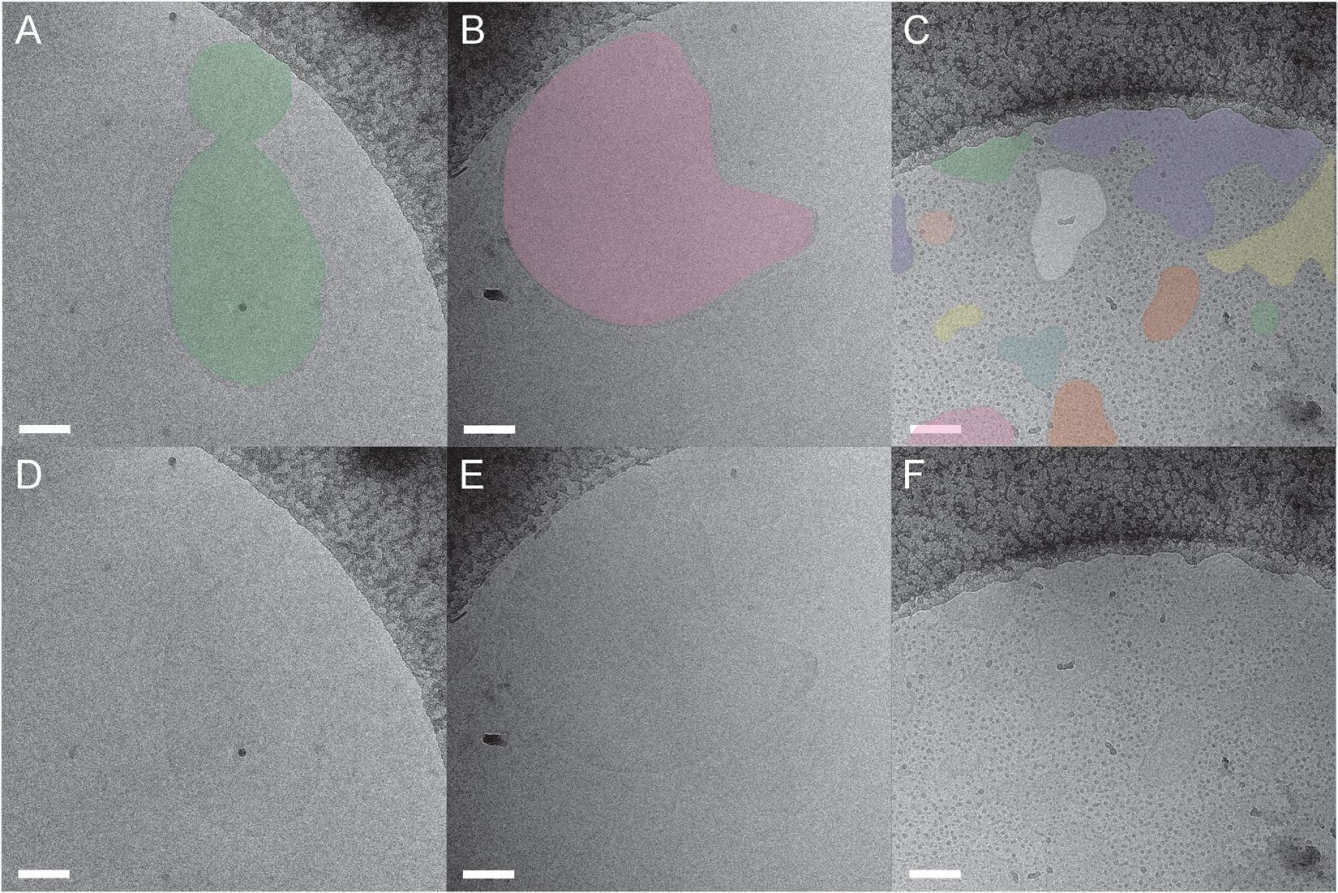
Cryo-EM images of sonicated non-crosslinked nanobubbles. (**A–C**) Disrupted nanobubbles are shown colorized for better visualization. Typically, disrupted nanobubbles are observed along the somewhat hydrophobic carbon support layer of the EM grid (dark gray). Both deformed sheet-like structures and dot-like objects are observed suggesting the instability of nanobubbles after sonication. (**D–F**) Non-colorized versions of panels A-C. Scale bars, 100 nm.

In contrast, cryo-EM images of ultrasound treated non-crosslinked NBs reveal significantly more disruption (Fig. 5). Not only are the electron dense cores missing, but also the nanobubbles themselves have been transformed into a heterogeneous mixture of deformed sheet-like structures and dot-like objects. The dot-like objects are <10 nm and could correspond to self-assembled Pluronic micelles^28–30^. The cryo-EM observation of a heterogeneous mixture is consistent with the DLS observation of a substantially broadened size distribution for non-crosslinked NBs (Fig. 2D). The observation that CL-NBs do not reform as liposomes or micelles after exposure to a high power ultrasound flash is likely due to the hydrophobic acrylamide component (NNDEA), which is not present in the non-crosslinked nanobubbles. Presumably, the acrylamide helps CL-NBs to retain their shape even after ultrasound disruption and gas release. Our finding of ultrasound disruption of non-crosslinked nanobubbles and subsequent reformation into micelles or large lipid sheets is in agreement with other studies that have investigated the fate of lipid bubbles after sonication^16,31,32^.

**Figure 6:**
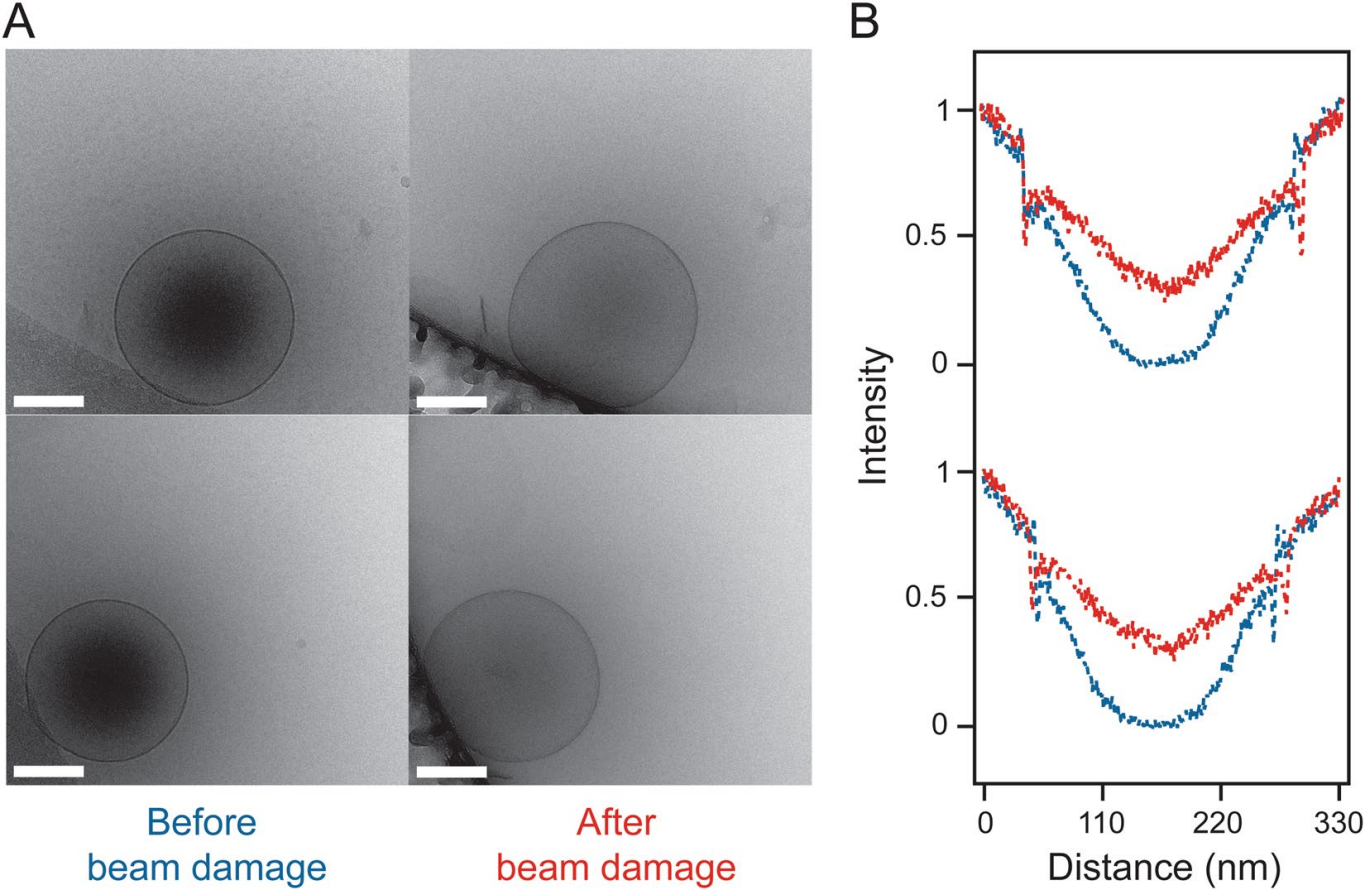
Effect of extended electron beam exposure on CL-NBs. (**A**) Representative CL-NBs imaged with a typical electron beam exposure (60 e^−^/Å^2^) (*left*) and after extended electron beam exposure (900 e^−^/Å^2^) (*right*). Scale bars, 100 nm. (**B**) Density plot analyses of these CL-NBs before (*blue*) and after extended electron beam exposure (*red*). The plots reveal a decrease in the density in the center of the nanobubbles suggesting loss of perfluorocarbon gas following extended electron beam exposure. The density of the surrounding frozen ice layer before and after extended electron beam exposure has been normalized to aid comparison.

While collecting cryo-EM images of CL-NBs we noticed that the high voltage (200 kV) electron beam of the transmission electron microscope had an effect on the dense cores. Upon extended electron beam exposure (~900 e^−^/Å), a gradual decrease in the central density within the nanobubbles is observed (Figs 6 and S3). It is well known in the cryo-EM field that electron irradiation breaks covalent bonds^33^. Therefore, we postulate that with sufficient electron beam exposure small holes are created in the lipid/polymer shell of the CL-NBs resulting in loss of the enclosed perfluorocarbon gas. The released gas is quickly dissipated due the high vacuum maintained in the transmission electron microscope. The gradual reduction in the density of the central cores of several CL-NBs can be observed with cryo-EM exposure series collected over ~40 s (Supplemental Movies S1–S3). Overall, the cryo-EM results presented here indicate that the dark centers observed in crosslinked and non-crosslinked nanobubble preparations are likely due to the encapsulated perfluorocarbon gas. Additionally, the CL-NBs can be disrupted with high-energy electrons, and this seems to result in release of the enclosed gas.

We considered whether the enclosed octafluoropropane gas liquefies during vitrification of the sample. Since the vitrification of the sample is rapid, we assume that the integrity of the outer pluronic/phospholipid layer remains intact. This is in analogy to cryo-EM studies of liposomes, in which the rapid cooling rate of vitrification ensures minimal perturbation to the sample^34,35^. If the pluronic/phospholipid layer remains intact, then we further assume that the enclosed gas cannot escape during vitrification. The diffuse and evenly distributed density observed in the nanobubble centers by cryo-EM (Figs. 3 and S2), suggests that the enclosed octafluoropropane remains in a gaseous state. If the octafluoropropane had liquefied during vitrification, we would expect to see condensed droplets inside of the nanobubbles.

Taken together, our DLS and cryo-EM results indicate that CL-NBs have increased stability compared to non-crosslinked NBs. Additional *in vitro* and *in vivo* studies have showed a 2-fold higher stability of CL-NBs as compared to non-crosslinked NBs without NNDEA^23^. This increased stability directly translates into longer blood circulation times, and therefore would be advantageous in applications where ultrasound contrast agents need to target cell surface markers and require additional circulation time. Likewise, CL-NB structural stability could be beneficial for drug delivery applications where increased cargo capacity and retention of drug without leakage may lead to improved therapeutic effects. However, additional experiments are needed to explore the benefits of CL-NBs for therapeutic applications.

## Conclusions

This work demonstrates the use of cryo-EM for imaging shell stabilized nanobubbles with a perfluorocarbon gas core. Several lines of evidence support our hypothesis that the dark cores observed in cryo-EM images of nanobubbles correspond to the encapsulated octafluoropropane gas. When CL-NBs are subjected to a high power ultrasound flash prior to creation of cryo-EM grids, the dense cores of the nanobubbles are lost. In addition, when CL-NBs are exposed to a 200 kV electron beam for several seconds, the density within the cores is observed to dissipate. Both the DLS and cryo-EM results presented here demonstrate that the polymer-stabilized CL-NBs have increased stability compared to non-crosslinked nanobubbles. The nanoscale ultrasound contrast agent field is rapidly developing. Quantitative structural data pertaining to the interaction of gas nanobubbles with ultrasound and evidence of their ultimate fate will be an essential component of future development and applications of nanobubbles in molecular imaging, as well as drug delivery. Cryo-EM is poised to be a key tool in providing structural data and facilitating nanobubble translation to clinical applications.

## Electronic supplementary material

Supplementary Information

Movie S1

Movie S2

Movie S3
