# Supplementary material for: Cryo-EM Visualization of Lipid and Polymer-Stabilized Perfluorocarbon Gas Nanobubbles - A Step Towards Nanobubble Mediated Drug Delivery
Source: Sci Rep. 2017 Oct 18;7:13517. doi: 10.1038/s41598-017-13741-1 (PMC5647366; doi:10.1038/s41598-017-13741-1)
Supplement: Supplementary file 1 — Supplementary Information [file 41598_2017_13741_MOESM1_ESM.pdf]

Electronic Supplementary Information for

Cryo-EM Visualization of Lipid and Polymer-Stabilized Perfluorocarbon Gas Nanobubbles - A Step Towards Nanobubble Mediated Drug Delivery

Christopher Hernandez[1*], Sahil Gulati[2.4*], Gabriella Fioravanti[1], Phoebe L. Stewart[2.4], Agata A. Exner[1,3]

Departments of Biomedical Engineering[1], Pharmacology[2], and Radiology[3], and Cleveland Center for Membrane and Structural Biology[4], Case Western Reserve University, Cleveland, Ohio USA

**These authors contributed equally*

Correspondence should be addressed to: agata.exner@case.edu and pls47@case.edu

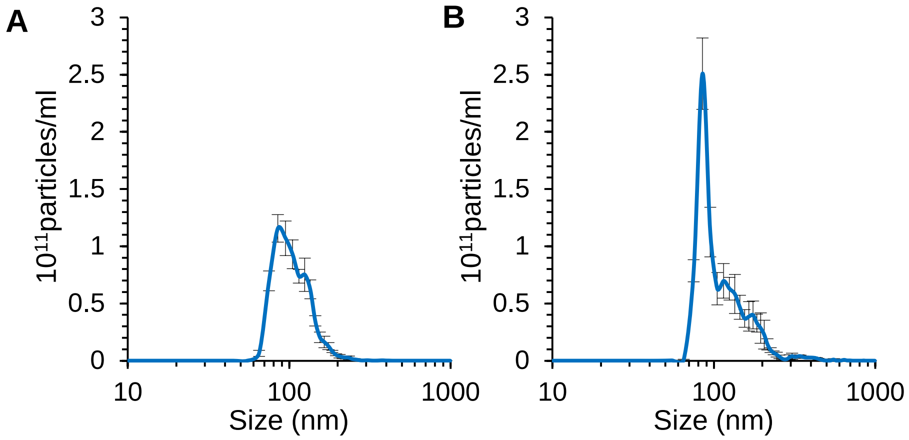

Figure S1. Representative plots showing nanobubble size distribution histogram and concentration from individual nanoparticle tracking analysis from (A) non-crosslinked nanobubbles and (B) crosslinked nanobubbles (CL-NBs).

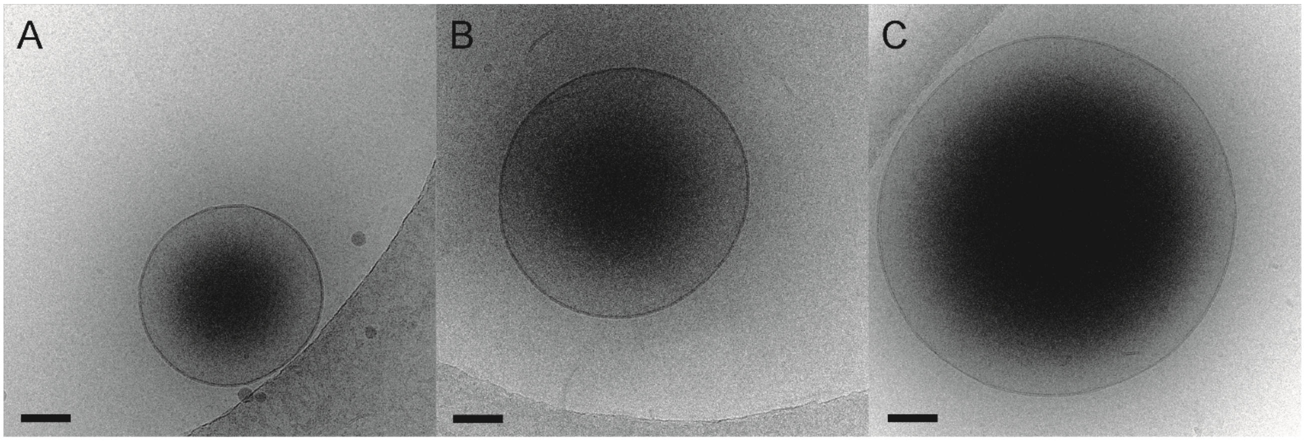

Figure S2. Cryo-EM images of non-crosslinked nanobubbles. (A) A single nanobubble close to the carbon support layer of the EM grid (dark gray, lower right-hand corner). (B-C) Images of intermediate-size and large nanobubbles. Note that the larger the diameter of the nanobubble, the darker its central core due to the increased path length for the electrons. Scale bars, 100nm.

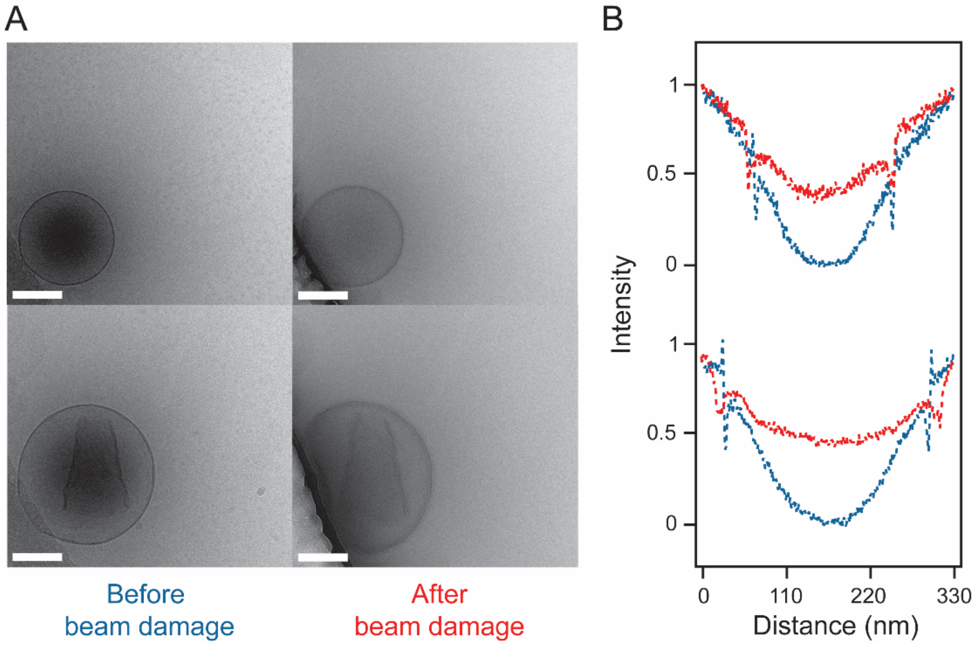

Figure S3. More examples of cryo-EM images showing the effect of extended electron beam exposure on CL-NBs. (A) Representative CL-NBs imaged with a typical electron beam exposure (60 $e^-/Å^2$) (*left*) and after extended electron beam exposure (900 $e^-/Å^2$) (*right*). Scale bars, 100nm. (B) Density plot analyses of these CL-NBs before (*blue*) and after extended electron beam exposure (*red*). The plots reveal a decrease in the density in the center of the nanobubbles suggesting loss of perfluorocarbon gas following extended electron beam exposure. The density of the surrounding frozen ice layer before and after extended electron beam exposure has been normalized to aid comparison.

MOVIE LEGENDS

Movie S1. Cryo-EM visualization of electron-beam induced damage to a crosslinked nanobubble (CL-NB). The movie shows a sequence of cryo-EM image frames each collected with an 800 ms exposure time over a total acquisition time of 40-45 s with a total electron dose of <900 $e^-/Å^2$. After extended electron beam exposure, the interior of the CL-NB gets lighter suggesting loss of the enclosed perfluorocarbon gas.

Movie S2. Cryo-EM visualization of electron-beam induced damage to a second crosslinked nanobubble (CL-NB). The movie shows a sequence of cryo-EM image frames each collected with an 800 ms exposure time over a total acquisition time of 40-45 s with a total electron dose of <900 $e^-/Å^2$. After extended electron beam exposure, the interior of the CL-NB gets lighter suggesting loss of the enclosed perfluorocarbon gas.

Movie S3. Cryo-EM visualization of electron-beam induced damage to a third crosslinked nanobubble (CL-NB). The movie shows a sequence of cryo-EM image frames each collected with an 800 ms exposure time over a total acquisition time of 40-45 s with a total electron dose of <900 $e^-/Å^2$. After extended electron beam exposure, the interior of the CL-NB gets lighter suggesting loss of the enclosed perfluorocarbon gas. In the last several frames of this movie, the frozen ice layer supporting the nanobubble is melting (see growing light gray circle in right-hand side of image).
